# Parasite clearance after malaria therapy: staying a step ahead of drug resistance

**DOI:** 10.1186/s12916-015-0486-1

**Published:** 2015-10-02

**Authors:** Harin A. Karunajeewa

**Affiliations:** Walter and Eliza Hall Institute of Medical Research, 1G Royal Parade, Parkville, Vic 3052 Australia; Western Health, Gordon Street, Footscray, Vic 3011 Australia

**Keywords:** Antimalarial, Artemisinin combination therapy, *Falciparum*, Malaria, Parasite clearance

## Abstract

The discovery and development of the artemisinin class of antimalarial drugs is one of the great recent success stories of global health. However, after at least two decades of successful use, resistance has finally emerged and appears to be spreading rapidly throughout South-East Asia in spite of our best efforts at containment. If this were also to occur in Africa, it would have disastrous implications for the continent subject to the world’s greatest burden of *Plasmodium falciparum*. The earliest indications of incipient artemisinin resistance may be a slowing of the rate at which parasites are cleared from the blood following treatment. The Worldwide Antimalarial Resistance Network have analysed data from 29,493 patients from 84 clinical trials in order to define the nature and determinants of early parasite clearance following artemisinin-based treatment in African populations. In doing so, they lay the foundation for systems intended to enable the earliest possible detection of emerging artemisinin resistance in Africa.

Please see related article: http://www.biomedcentral.com/1741-7015/13/212

## Background

The last decade has seen unprecedented improvements in global malaria control. Current estimates suggest that, worldwide, malaria-attributed deaths (that occur mostly in young children) have fallen by 47 % since 2000 [1]. Therefore, many millions of today’s young people owe their lives to this progress. A key to this success has been improved availability of safe, highly effective antimalarial drugs. In particular, the rediscovery of the ancient Chinese herbal medicine, artemisinin, has been transformative since it first became widely used in the 1990s [2]. This remarkable drug class is distinguished by affordability, an excellent safety profile, and potent parasiticidal activity that manifests as a rate of malaria parasite “log kill” an order of magnitude greater than that of previously available drugs. Very early on, artemisinins were recognized as such a precious resource that strenuous efforts were advocated to protect them from the ravages of drug resistance [3]. This underpinned the rationale for deployment in combination with a second, longer acting partner drug, leading to artemisinin-based combination therapy (ACT). ACTs are now the cornerstone of global malaria treatment policy, being recommended by WHO as the first-line drugs of choice for most of the estimated 198 million annual global cases of malaria illness [4]. They are recognized as vital tools for over 35 countries that have now set nationwide elimination as the explicit objective of their malaria programs [1]. ACTs may therefore yet play a significant role in the audacious goal of eventual complete global malaria eradication [5].

Sub-Saharan Africa carries the world’s greatest burden of *Plasmodium falciparum* and has seen some of the world’s greatest gains in malaria control, including an estimated 54 % reduction in mortality since 2000 [1]. However, optimism here has been tempered firstly by the recent advent of high-level insecticide resistance in African mosquito vectors and secondly by the alarming emergence and spread of artemisinin-resistance in South-East Asia [1, 6]. Were artemisinin resistance to also arise in Africa, the consequences could be catastrophic. This could occur if South-East Asian *P. falciparum* strains found their way to Africa, or as is perhaps more likely, if resistance were to arise *in situ* in Africa as a separate independent event. The lessons from South-East Asia are sobering. Delayed early parasite clearance was first reported in the Pailin region of Western Cambodia in 2008 [7]. By 2014, gene mutations associated with this resistant phenotype were already present in five South-East Asian countries and appeared close to encroaching on the Indian sub-continent [6]. Resistance has also now developed in ACT partner drugs, presumably because they effectively became unprotected monotherapies once the artemisinin component had been compromised [8, 9]. This has occurred despite unprecedented levels of international financial assistance being mobilized to attempt to contain the spread of resistance from as early as 2008. Robust mechanisms for early detection and prompt response are necessary to avert such a scenario unfolding in Africa.

## Early detection of antimalarial drug-resistance

**Fig. 1:**
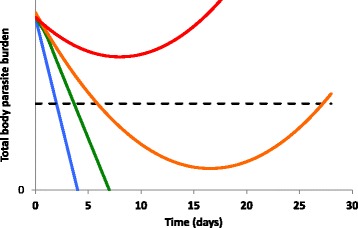
Relationship between early parasite clearance and drug resistance. This hypothetical example shows how the parasite clearance curve might change as drug resistance progressively compromises an artemisinin-based combination therapy (ACT). Parasite clearance curves are shown for fully sensitive (*blue*), early resistance (*green*), established resistance (*orange*), and advanced resistance (*red*) scenarios. The earliest event in the progression of resistance is delayed early parasite clearance – but drug activity may still be sufficient to clear the total body parasite burden and achieve cure. As resistance becomes established, initial parasite killing may be sufficient for parasitemia to fall below the threshold of microscopic detectability (*dashed line*) but not to eliminate the entire body parasite burden – leading to recrudescence and late treatment failure. Complete failure to clear parasites (early treatment failure) will only occur once resistance to both drugs in an ACT combination has become very advanced

Early detection of drug resistance in malaria is problematic. *In vitro* parasite culture-based assays are generally poorly predictive of *in vivo* drug susceptibility phenotype, especially for the artemisinin derivatives. An exception is a “ring stage assay” used to characterize the South-East Asian artemisinin-resistant phenotype [10]. However, future foci of artemisinin resistance may arise through alternative, as yet unknown, biological pathways so may not be amenable to detection using this assay, nor to molecular methods for detecting associated mutations in the Kelch-13 propeller gene [11]. Therefore, in the foreseeable future, early detection of future foci of artemisinin resistance will probably still rely on “old-fashioned” methods of characterizing drug susceptibility by post-treatment clinical evaluation. The South-East Asian experience demonstrates the importance of early parasite clearance as the earliest sign of incipient artemisinin resistance (Fig. 1).

## Methods for measuring parasite clearance

Many methods have been used to define parasite clearance kinetics in the clinical trial setting. These include the time taken for parasitemia to become undetectable by microscopy (parasite clearance time), to decline by 50 % or 90 % of baseline and applying mathematical equations to define the slope of the parasite clearance curve [12, 13]. However, these all rely on very frequent (e.g. every 4 hours) blood slide microscopy and are impractical to implement in the field. By contrast, defining the proportion of a population sample with microscopically detectable parasitemia (the parasite positivity rate; PPR) at a given time is a more practical, albeit less sensitive index. The current WHO standardized 28-day protocol for *in vivo* evaluation of treatment response requires microscopy at days 2 and 3, and therefore these are especially feasible times to measure the PPR [14]. The approach adopted by the Worldwide Antimalarial Resistance Network (WWARN) investigators therefore represents a pragmatic strategy, well-suited to future operational deployment.

## Factors that influence parasite clearance

**Fig. 2:**
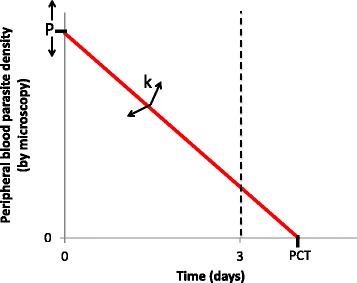
The parasite positivity rate (PPR) as a parasite clearance metric. The PPR at day 3 is defined by the proportion of a population with detectable parasitemia 3 days following treatment initiation. Therefore, for each individual, it reflects a binary outcome according to whether the X-intercept of the parasite clearance curve (also defined as the parasite clearance time) occurs before or after day 3 (*dashed line*). Whether this occurs or not will depend on the Y intercept (P: the parasite density at treatment commencement) and the gradient of the parasite clearance curve (k: the rate at which parasites are cleared). k will be determined by intrinsic parasite sensitivity (and therefore decrease when drug resistance develops) but may also be affected by pharmacological (e.g. pharmacokinetic variability) and host factors (acquired malaria-specific immunity will augment parasite killing and therefore increase the gradient)

Figure 2 shows how PPR will be dependent on two main factors, namely (1) how high the total body parasite burden is prior to treatment and (2) the rate of parasite clearance. Unfortunately, the relationship between parasite clearance rate and drug-resistance may be confounded by factors other than parasite drug-susceptibility. These include pharmacologic factors (including pharmacokinetic variability and patient non-compliance) and host factors, specifically pre-existing malaria-specific immunity that can augment parasite killing. In a research article published in *BMC Medicine* [15], the WWARN investigators identify a number of factors associated with the PPR at day 3, many of which (age, fever, severe anaemia, and low transmission settings) probably represent separate proxies for malaria-specific immunity. The gradual acquisition of malaria immunity following repeated infections mean that, in high transmission settings, population immunity is higher so parasite clearance should be more rapid. The clinico-epidemiologic context should therefore be considered when determining the all-important threshold values for triggering a more intensive investigative response. The authors make an excellent case that existing WHO-recommended thresholds may be insufficiently sensitive for this purpose if applied in high transmission settings in Africa, risking the delayed identification of future new foci of artemisinin resistance.

## Conclusions

As the modern global malaria eradication agenda continues to gain momentum, we are likely to see even more intense malaria control activities that aim to capitalize on recent successes. Increased antimalarial drug use as part of these efforts could see *P. falciparum* in Africa subject to the highest levels of selective pressure in its entire evolutionary history. It seems prudent, therefore, to consider the prospects of artemisinin resistance in Africa as a matter of “when” rather than “if”. The data presented by the WWARN investigators provides some reassurance that this spectre is not yet upon us. However, some included studies were conducted as early as 1999 and it is possible that, somewhere in Africa, events have already overtaken these data. Others have warned against over-reliance on parasite clearance for detecting drug resistance, noting that, because malaria-specific immunity has such a large effect on parasite clearance, it may “mask” any changes due to drug resistance from being discernible, making parasite clearance metrics insensitive surrogates for drug resistance [16]. Nonetheless, the work of the WWARN group represents an impressive effort in international collaboration and has laid the foundations for a global early warning system designed to detect early signs of artemisinin resistance using a simple, easily defined parameter that can be generated by existing operational protocols. We can only hope that the next time artemisinin resistance arises, we can lock the stable door before the horse has a chance to bolt.

## Abbreviations

ACT: Artemisinin-based combination therapy
PPR: Parasite positivity rate
WWARN: Worldwide Antimalarial Resistance Network
